# The second decade of synthetic biology: 2010–2020

**DOI:** 10.1038/s41467-020-19092-2

**Published:** 2020-10-14

**Authors:** Fankang Meng, Tom Ellis

**Affiliations:** 1 Imperial College Centre for Synthetic Biology, Imperial College London, London, SW7 2AZ UK; 2 Department of Bioengineering, Imperial College London, London, SW7 2AZ UK; 3 Wellcome Trust Sanger Institute, Wellcome Genome Campus, Hinxton, Cambridge CB10 1SA UK

**Keywords:** Genetic engineering, Synthetic biology

## Abstract

Synthetic biology is among the most hyped research topics this century, and in 2010 it entered its teenage years. But rather than these being a problematic time, we’ve seen synthetic biology blossom and deliver many new technologies and landmark achievements.

In 2020 synthetic biology turned 20 years old. It’s first decade saw some impressive research papers, lots of visionary thinking and unprecedented excitement, but its second decade—from 2010 to 2020—was when the hype really needed to be replaced by some real achievements. So how has it done?

The decade got off to a great start. Looking back at 2010, the biggest synthetic biology story of the year was the complete synthesis of a working bacterial genome by a team at the J. Craig Venter Institute (JCVI)^1^. A landmark achievement that showed that DNA synthesis and DNA assembly could be scaled to megabase size, delivering on some of the biggest ambitions from the start of the century. However, just scaling DNA construction would not be enough to deliver the field’s many other ambitions. 2010 also saw the publication of ‘*Five Hard Truths for Synthetic Biology*’ a critical article that examined how the lack of progress on engineering ambitions was slowing efforts to deliver on promises of reliability, standardisation and automated design^2^. These were indeed problems for the field, and were highlighted in one of the first synthetic biology papers in *Nature Communications* which showed a robust genetic logic gate failing when moved into different *E. coli* strains^3^. Could hard biological problems such as context, noise, burden and cross-reactivity really be solved to allow us to engineer cells like we wire-up electronic circuits?

Well thanks to a lot of challenging technical biology and biological engineering work undertaken by many in the field, but especially MIT’s Chris Voigt, the answer to this was yes. In 2016 Nielsen et al., published Cello, a remarkable end-to-end computer aided design system for logic circuit construction in *E. coli*^4^. Of all the papers in the last decade, this is probably the most satisfying for hardcore synthetic biologists as it realises so much of the promised engineering of biology and does so through standardisation, characterisation and automated design. It’s no coincidence that in the years preceding this paper Voigt’s team worked tirelessly on delivering so many other foundational papers on *E. coli* synthetic biology, giving us algorithmic design of genetic parts, and professional characterisation of part libraries. While it is easy to focus on the many big landmark achievements of synthetic biology (Fig. 1), what has really helped the field deliver on the hype more than anything else has been a lot of hard technical work to improve our design and understanding of genetic parts alongside innovation and the discovery of new technologies that let us write, build, edit and share DNA code better than ever (Fig. 2).

**Fig. 1:**
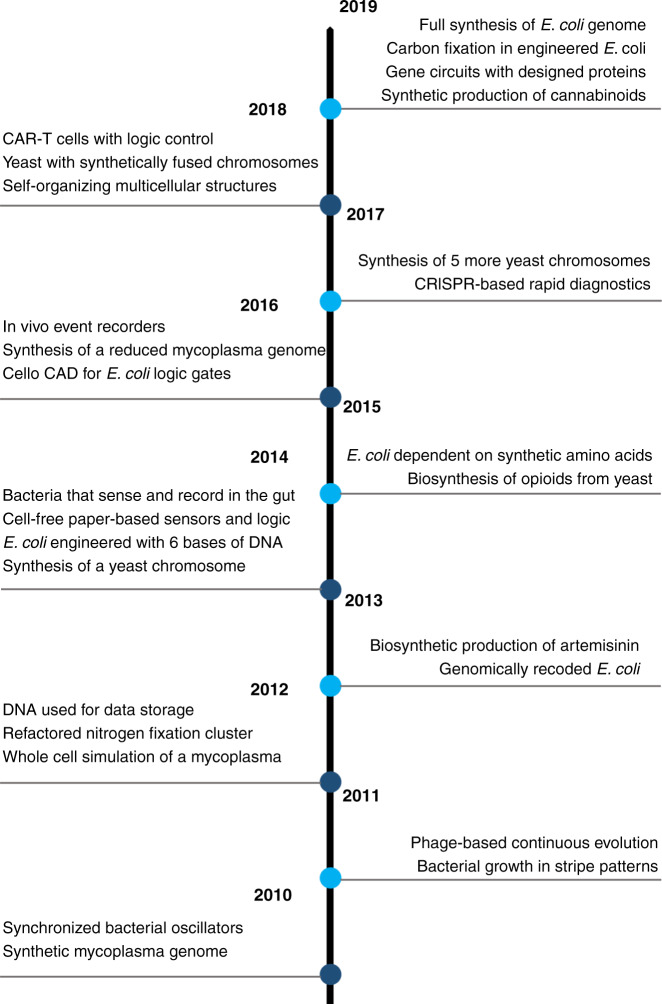
Landmark research achievements of synthetic biology from 2010 to 2020. A timeline is shown for the decade with brief summaries of some of the key research milestones published for each year.

**Fig. 2:**
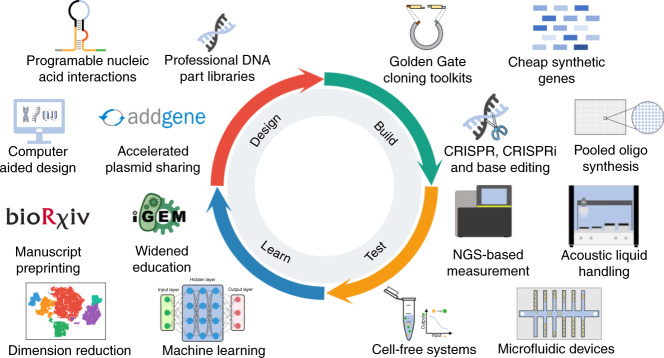
New enabling technologies and ways of working that have accelerated the design-build-test-learn cycle of synthetic biology in the last decade. Diagram shows the engineering cycle used in synthetic biology (centre) with cartoons illustrating some of the key technologies and ways of working that now help accelerate each phase of the cycle.

Indeed, looking back 10 years what is most striking is how the methods and tools have changed for those engineering life. Many groups were still reliant on the iGEM competition for exchanging their DNA parts. These could only be assembled serially by BioBrick cloning, and performance data on these parts was rarely reliable. Building a genetic construct was slow lab work and typically resulted in failure when added into a cell. Methods to fix a DNA construct once inside a cell were severely lacking and in high demand. It’s no surprise therefore, that synthetic biology groups were the first to pounce on gene editing technologies like CRISPR as they appeared in 2011 and 2012. Since 2006, George Church’s lab had been pursuing a project to mutate every TAG stop codon to TAA in *E. coli* to free up a codon for an extra amino acid^5^, and so unsurprisingly they were very interested in developing any technology that can precisely alter DNA inside a cell. His group and his former postdoc published the first two papers in 2013 showing CRISPR being used in eukaryotic cells and developed many further innovations of CRISPR in the following years, including CRISPR-based gene drives. Synthetic biology groups in California were also quick to turn CRISPR into more than just a cutting tool, inventing the now ubiquitous *dCas9* as a programmable binder of DNA to enable customisable gene regulation^6^.

While there’s no doubt that CRISPR was the breakthrough of the decade in biosciences, it’s perhaps its forerunner TALENs (TAL-Effector Nucleases) that deserve more credit in revolutionizing how synthetic biology changed in the past 10 years. The promise of modular, programmable binding of any DNA sequence and an in vivo gene editing method lured many towards this new technology in 2011. But the large highly-repetitive nature of TALENs was a slap in the face for anyone used to standard DNA cloning methods, PCR or even Gibson Assembly. If you wanted to work with TALENs you needed to either buy a lot of synthesised DNA, become an expert in robot-based DNA assembly automation, or get your hands on a Golden Gate Assembly TALEN cloning toolkit from Addgene. Fortunately for synthetic biology, many people in the field tried one, two, or even all three of these things and soon began to see how much faster DNA assembly can proceed. Golden Gate DNA Assembly now rightly dominates the field and the many modular cloning (MoClo) toolkits and genetic part libraries shared via Addgene have transformed the way people work and build upon each other’s findings. Companies and academic institutions have set up dedicated facilities for automated DNA cloning and perhaps most importantly, DNA synthesis costs have fallen to the point where ordering a custom-made gene is now often more cost-efficient than trying to clone it. Such an enabling drop in price cannot be underestimated for how it changes people’s way of working for the better.

The drop in cost for gene synthesis can mostly be attributed to new methods for printing thousands of oligonucleotides in parallel on chips to make ‘oligo pools’ and teaming this with next generation sequencing (NGS) as a much more cost-effective method for validating assembled DNA. These two technologies also opened the door to a major change in the way people worked in synthetic biology, suddenly making it cost effective to design, build and measure in parallel hundreds of thousands of genetic designs in one experiment^7^. If you can tie the output of your genetic design to an NGS-compatible measurement (e.g. barcoded RNAseq) then data analysis becomes your new bottleneck, not design and assembly as before. This has led to a major shift in the relationship between mathematical approaches and biology in synthetic biology over the decade. When making and testing DNA constructs was slow and expensive in the 2000s, mathematical modelling was valuable to predict successes and failures, and to narrow down the design space. Now this approach is rarely needed, and mathematical analysis instead finds its value in statistical analysis of large data sets and using this to learn how to design DNA.

High-power computation also opened up new frontiers in what can be modelled and predicted in the last 10 years. Rational design of proteins, spearheaded by David Baker’s group, came on leaps and bound, and ended the decade as part of gene circuitry in living yeast cells^8^. The first ever whole cell model, for *Mycoplasma genitalium*, was released, enabling simulation of the effects of hundreds of genes through a cell cycle^9^. This helped inform JCVI’s project towards a minimal genome, which delivered a further landmark in 2016 with the impressive engineering of a bacteria with a minimized synthetic genome^10^.

Synthetic genomics also moved into eukaryotes with the international Sc2.0 consortium constructing highly-modified, yet fully-functional synthetic versions of Baker’s yeast chromosomes^11^. *E. coli* also ended the decade with a synthetic genome, redesigned and constructed to remove all use of 3 of the 64 codons of the genetic code^12^. Such recoding enables cells to be engineered to readily insert non-standard amino acids into proteins as desired. This was pioneered by the Church lab’s mutation-based approach that reduced *E. coli* to using only 63 codons earlier in the decade, demonstrating expansion of the genetic code^5^. Expansion of this code was also achieved in *E. coli* by addition of bases of DNA beyond just A, T, C and G.

DNA also became a way to store data, initially just in vitro via chemical synthesis, but then also in cells via ‘molecular recorder’ genetic systems that use recombinases or CRISPR to modify DNA as cells grow, divide and change their gene expression. Sensing and recording in cells even went into the body, with gut bacteria sensing and reporting on events inside mice. Engineered probiotic bacteria were made that detect cancer in urine and others turned them into therapies, correcting metabolic disorders and sensing and destroying pathogens. The hottest cell-based therapies in the pharma industry, cancer-targeting CAR-T cells, also started to be equipped with sensing and logic devices from synthetic biology. Sensing and logic with synthetic biology also found further healthcare applications, including paper-based biosensors that could be rapidly designed to detect RNA from pathogens like Ebola and Zika^13^. These sensors, along with other recent applications, were enabled by new modular ways to design complex nucleic acid interactions, such as toehold switches, and by a new ‘cell-free’ way to do synthetic biology, using lysates from cells as customisable and accessible alternatives to in-cell engineering^14^.

Healthcare has now arguably replaced metabolic engineering as the go-to for synthetic biology applications, but that has not stopped progress in this area too. Academic achievements include engineering cells to fix CO_2_ and nitrogen, and getting yeast to make opioids and cannabinoids. Biofoundries have been established at many institutes and can demonstrate rapid engineering of cells for biosynthesis of dozens of different molecules^15^. Of course, much of the work in using synthetic biology for metabolic engineering now happens at companies like Amyris, Genomatica, Ginkgo and Zymergen.

Looking back on the decade, the many research landmarks and new directions for synthetic biology are indeed very impressive, but as synthetic biology researchers it’s the advances in enabling technologies that excite us the most as these unlock what can come next. However, if we’re to look for the single biggest achievement of the decade that justifies the hype of the field back in 2010, then we can look no further than the proliferation and valuations of the hundreds of synthetic biology companies around the world. A multibillion dollar industry now exists that makes chemicals, drugs, proteins, probiotics, sensors, fertilisers, textiles, food and many other things from engineered cells. And these are not existing companies just buying into synthetic biology, but companies founded, led and grown by the postdocs, PhDs and iGEM students who researched at the bench and in most cases have worked in this field their entire adult lives.
